# Supplementary material for: Investigating Morphological and Physiological Responses to Stress in Begonia semperflorens
Source: Int J Mol Sci. 2025 Apr 9;26(8):3514. doi: 10.3390/ijms26083514 (PMC12026712; doi:10.3390/ijms26083514)
Supplement: Supplementary file 1 [file ijms-26-03514-s001.zip › ijms-3506251-supplementary.pdf]

International Journal of
Molecular Sciences

MDPI

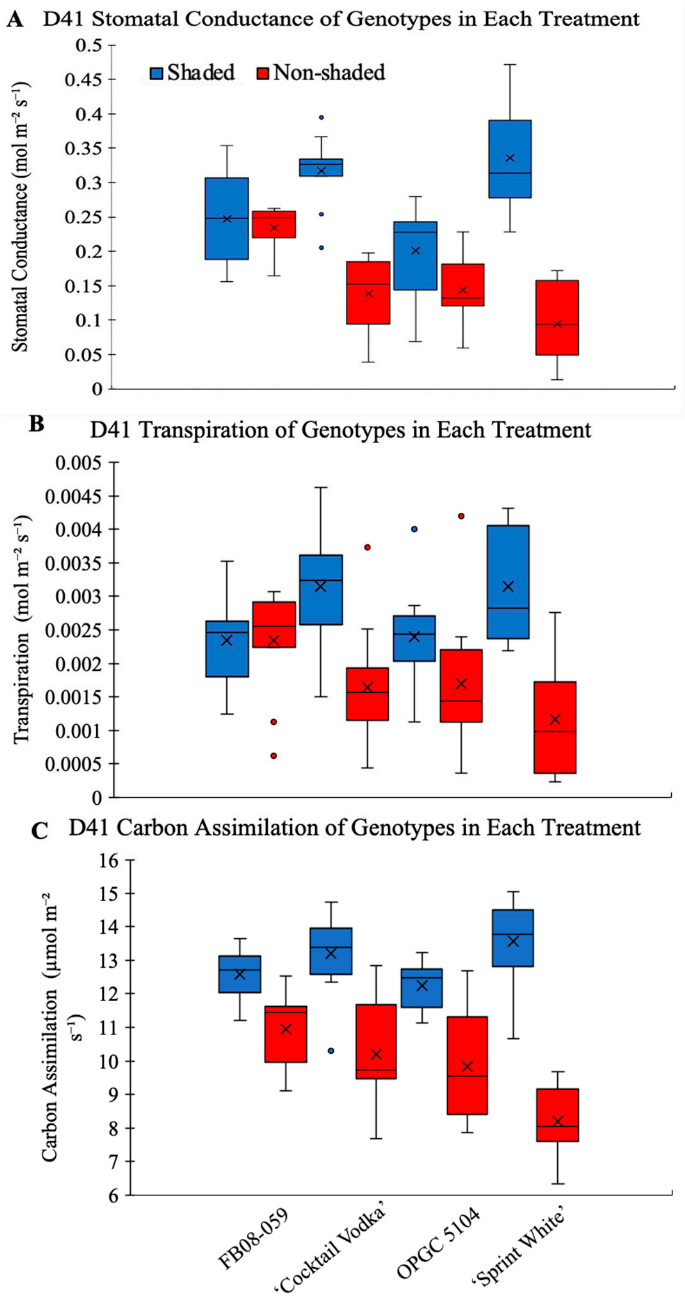

Supplemental Figure S1. Day 41 measurements of stomatal conductance (A), transpiration (B), and Carbon Assimilation (C) in the shaded and non-shaded conditions.

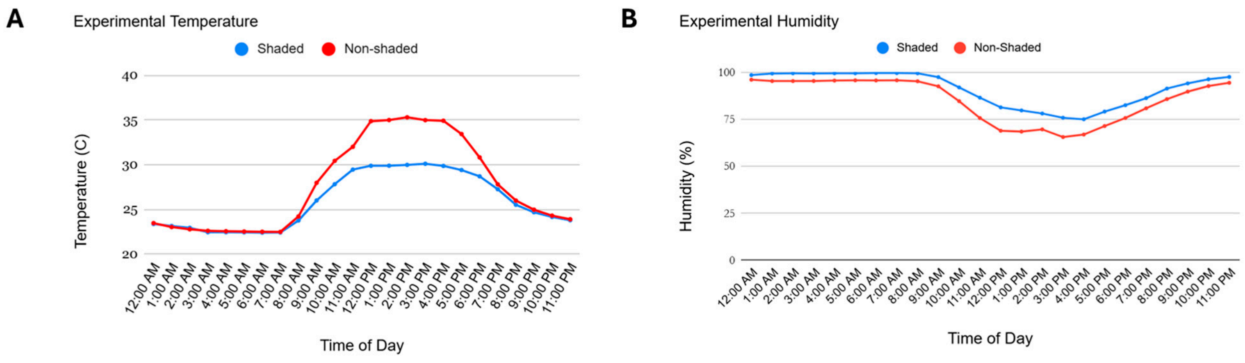

Supplementary Figure S2. Environmental conditions during the experiment: (A) Temperature profile under shaded and non-shaded conditions over a 24-hour period. The non-shaded condition resulted in significantly higher midday temperatures, peaking at approximately 37°C, compared to the shaded condition, which remained below 32°C. (B) Relative humidity variations throughout the day. The non-shaded condition experienced lower humidity levels, particularly during peak daylight hours, whereas the shaded environment maintained consistently higher humidity.
